# Innovative approaches to greywater micropollutant removal: AI-driven solutions and future outlook

**DOI:** 10.1039/d5ra00489f

**Published:** 2025-04-22

**Authors:** Mohamed Mustafa, Emmanuel I. Epelle, Andrew Macfarlane, Michael Cusack, Anthony Burns, Mohammed Yaseen

**Affiliations:** a School of Computing, Engineering & Physical Sciences, University of the West of Scotland Paisley PA1 2BE UK mohammed.yaseen@uws.ac.uk; b School of Engineering, Institute for Infrastructure and Environment, The University of Edinburgh Edinburgh EH9 3JL UK; c ACS Clothing 6 Dovecote Road Centralpark ML1 4GP UK

## Abstract

Greywater constitutes a significant portion of urban wastewater and is laden with numerous emerging contaminants that have the potential to adversely impact public health and the ecosystem. Understanding greywater's characteristics and measuring the contamination levels is crucial for designing an effective recycling system. However, wastewater treatment is an intricate process involving significant uncertainties, leading to variations in effluent quality, costs, and environmental risks. This review addresses the existing knowledge gap in utilising artificial intelligence (AI) to enhance the laundry greywater recycling process and elucidates the optimal treatment technologies for the most prevalent micropollutants, including microplastics, nutrients, surfactants, synthetic dyes, pharmaceuticals, and organic matter. The development of laundry greywater treatment technologies is also highlighted with a critical discussion of physicochemical, biological, and advanced oxidation processes (AOPs) based on their functions, methods, associated limitations, and future trends. Artificial neural networks (ANN) stand out as the most prevalent and extensively applied AI model in the domain of wastewater treatment. Utilising ANN models mitigates certain limitations inherent in traditional adsorption models, particularly by offering enhanced predictive accuracy under varied operating conditions and multicomponent adsorption systems. Moreover, tremendous success has been recorded with the random forest (RF) model, exhibiting 100% prediction accuracy for both sessile and effluent microbial communities within a bioreactor. The precise prediction or simulation of membrane fouling behaviours using AI techniques is also of paramount importance for understanding fouling mechanisms and formulating efficient strategies to mitigate membrane fouling.

## Abstract 2

Greywater constitutes a significant portion of urban wastewater and is laden with numerous emerging contaminants that have the potential to adversely impact public health and the ecosystem.

## Graphical abstract

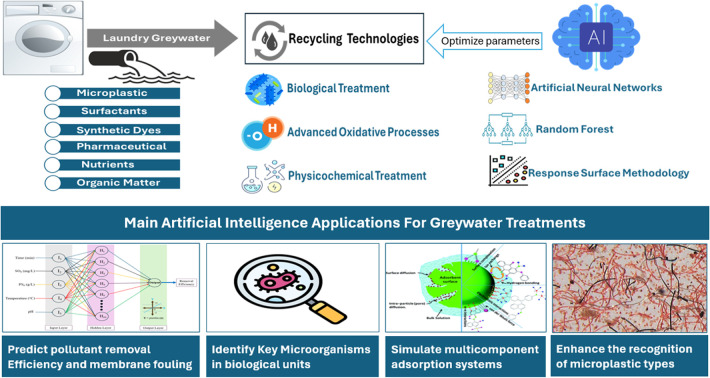

## 1. Introduction

Natural water recycling processes are vital for life on Earth. However, as the demand for water grows due to increasing population and industry requirements, these natural methods may not keep up with the rate at which water is contaminated and disposed of. For instance, a typical laundry facility with a processing capacity of 10 metric tons of linen generates 150 cubic meters of wastewater each day; it takes approximately 15 litres of fresh water to process a kilogram of linen in a commercial laundry. Industrial laundry wastewater constitutes approximately 10% of the total urban wastewater produced.^1,2^ While the demand for water continues to rise, freshwater resources per capita are projected to decrease by around 22% for the period from 2000 to 2025.^3^ This decline in freshwater availability is a critical issue that calls for immediate action, prompting the UN to prioritize water sustainability (SDG6).^4^ Wastewater treatment plants aid but cannot meet demand alone; industrial users must contribute to recycling. Understanding the characteristics of wastewater and measuring the level of contamination is crucial for designing an effective recycling system.

Traditional greywater treatment employs physical, chemical, biological and advanced oxidation processes that typically necessitate continuous monitoring and manual adjustments. Recent advancements in artificial intelligence (AI) and machine learning present novel opportunities for real-time monitoring, predictive analytics, and optimisation of treatment systems. However, the integration of AI in greywater micropollutant removal remains in its infancy, with challenges such as data availability, model accuracy, and scalability. Therefore, there is a pressing need to explore innovative AI-driven strategies for effective micropollutant removal, assess their feasibility, and outline future directions for sustainable and intelligent greywater treatment. This comprehensive review investigates the current knowledge gap in applying artificial intelligence (AI) to optimise the recycling of laundry greywater and identifies the most effective treatment technologies for common micropollutants, including microplastics, nutrients, surfactants, synthetic dyes, pharmaceuticals, and organic matter. Furthermore, it critically examines the development of laundry greywater treatment technologies, focusing on physicochemical, biological, and advanced oxidation processes (AOPs) by analyzing their mechanisms, methodologies, limitations, and emerging trends. There are few studies that focus on determining the most suitable greywater treatment method for specific pollutants under varying conditions or discussing laundry greywater recycling process in wholistic approach that includes both advances in treatment technologies and AI-driven solutions.

### 1.1. Wastewater classification

Human activities can cause various levels of damage to the used water. For instance, in domestic use, the produced wastewater can be categorised mainly to blackwater that is highly polluted with human waste and greywater which is less contaminated and includes predominantly detergents.^5^ The term greywater indicates the change of water colour to grey during storage or even before any storing period, such as when the water is generated during laundry operations. However, this term is often used to refer to wastewater produced from domestic activities, except for that generated from toilet seats.^6,7^ This exception is because black water generated from toilet seats has a high concentration of organic matter, nutrients, and pathogens.^8^ Bodnar *et al.*^9^ classified laundry and cooking wastewater as dark greywater and bath wastewater as light greywater. As shown in Table 1, light greywater contains various types of soaps, body care chemicals and some traces of urine and excrement. While dishwashing detergents, oil, grease, and food remains are common constituents found in kitchen greywater. On the other hand, laundry greywater mainly contains soaps, bleaches, oils, paint, solvents, and non-biodegradable fibres from clothing. Furthermore, laundry greywater is often hot saline water with a high pH level and includes elevated concentrations of nitrate, phosphate, and sodium.^10^

**Table 1:** Greywater sources and their constituents^10,11^

Light greywater		Dark greywater	
Wash basin	Bathroom	Laundry wastewater	Kitchen sinks
Body care chemicals, toothpaste, soaps, hair, skin cells and shaving waste	Shampoo, lint, body fats, hair, sand/clay, traces of urine and excrement	Fibres from clothing, paint, oils, soaps, bleaches and solvents	Oil and grease, food remains and dishwashing detergents

### 1.2. Wastewater pollutants and laboratory tests

**Fig. 1:**
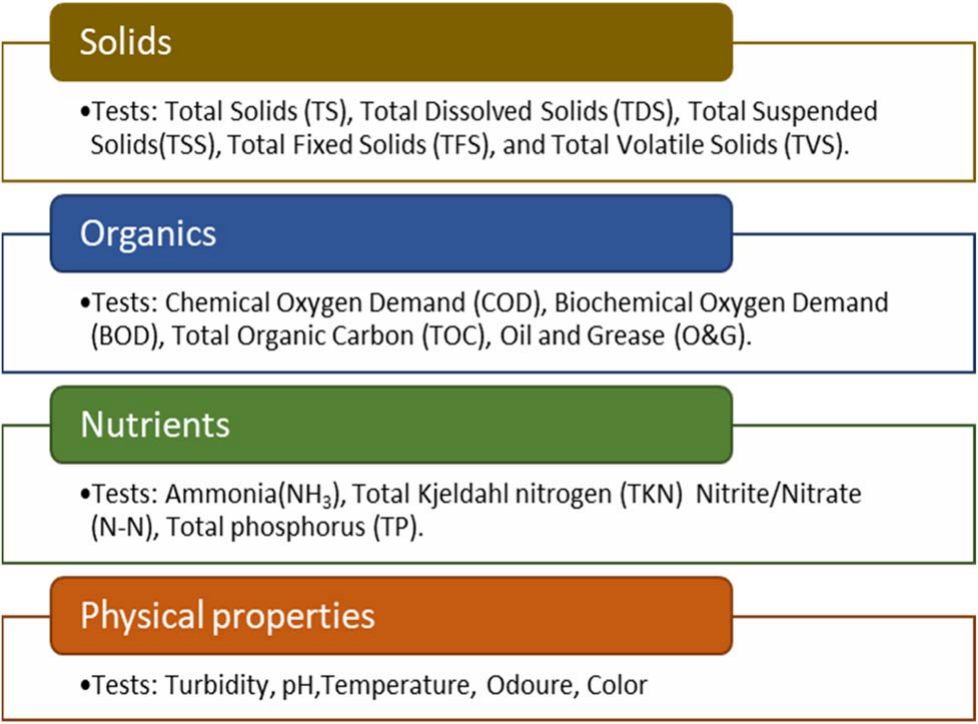
Laboratory tests and analytical categories of wastewater.

Pollutants in wastewater exist as solids, liquids, gases, or mixtures and are classified by solubility as dissolved or undissolved. Undissolved pollutants include food residues, human waste, oils, suspended solids, and microorganisms. Dissolved pollutants form anions, cations, or complex compounds, while certain gases react with wastewater, generating pollutants such as carbon dioxide, nitrogen dioxide and ammonia.^12,13^ Pollutants are further categorized by chemical composition and origin as organic, inorganic, volatile, non-volatile, biodegradable, refractory, or of animal, mineral, or vegetable origin.^14^ Wastewater quality is assessed by measuring organic matter, particulate solids, nutrients, and physical properties. As shown in Fig. 1, the evaluation of these four types of measurements or analytical categories can be achieved with various combinations of laboratory tests.^15^ For instance, chemical oxygen demand (COD), biochemical oxygen demand (BOD), total organic carbon (TOC) and oil and grease (O&G) tests, which identify the concentration of the carbon-based compounds in the tested sample, are often performed to indicate the level of organic pollutants. Additionally, total suspended solids (TSS), total dissolved solids (TDS), total solids (TS), total fixed solids (TFS), and total volatile solids (TVS) are all measurements of particulate solids that can be found in wastewater. There are no solid boundaries between these analytical categories; for example, organics in a wastewater sample BOD will also be represented in the spectrum of solids as suspended TSS or dissolved TDS particulates. Moreover, the concentration of nutrients, including nitrogen, phosphorous, and sulfate, can be measured to help determine the rate of eutrophication. In addition, measuring the turbidity, hardness, odour, pH, colour, and temperature are used to evaluate the physical properties of the wastewater samples.^8^

The remainder of this review is systematically organised into five subsequent sections. The first section presents a comprehensive overview of laundry wastewater, encompassing its fundamental characterisation parameters, predominant micropollutants, optimal treatment methodologies, and an assessment of microplastics as an emerging contaminant. The second section delves into the advancement of greywater treatment technologies, offering a critical evaluation of physicochemical, biological, and advanced oxidation processes (AOPs), with an emphasis on their underlying mechanisms, methodologies, inherent limitations, and novel innovations. The third section examines AI-driven solutions in greywater treatment, elucidating the integration of artificial intelligence within these technologies. The fourth section addresses the limitations and future trends of the field, discussing the challenges associated with AI models implementation in real-world applications, their applicability in laundry greywater treatment, and prospective research directions. Finally, the concluding section synthesises the key findings presented throughout the review.

## 2. Laundry wastewater

### 2.1. Laundry wastewater properties

The properties of laundry greywater and the level of contamination depend on the laundry wastewater generating sources. For instance, while domestic laundry greywater includes fat, oil, grease, and dirt in addition to soap and soda, hospitals' laundry greywater can contain traces of medicine drugs and chemical products, which can pose a public health threat by increasing the environmental antimicrobial resistance.^16^ Furthermore, greywater produced in commercial laundries often includes dyes, soaps, phosphate-based detergents, peroxide-based bleaches (*e.g.* sodium percarbonate), and surfactants, including anionic surfactants like linear alkylbenzene sulfonate (LAS) and non-ionic surfactants such as nonylphenol ethoxylates (NPEOs).^17,18^Table 2 summarises the results of analysing laundry wastewater collected from various sources, which include households, industries, and hospitals.^19^ The main factors that can cause variations in laundry greywater characteristics are the number and habits of the people using the service, as well as the washing behaviour which includes average load size, water temperature, the composition of the used cleaning products, and the number of washing cycles such as prewash, main wash and rinsing. Washing machine type can also play a role in the final characteristics of the greywater and the level of water consumption.^21,22^

**Table 2:** Characteristics of domestic, industrial and hospital Laundry wastewater.^19,20^

Parameters	Domestic laundry	Industrial laundry	Hospital laundry
pH	9.3–10	9–11	11.4–11.6
EC^a^ (mS cm^−1^)	0.190–1.400	0.640–3.000	0.808–2.000
TDS (mg L^−1^)	400–6000	640–3000	456–800
TSS (mg L^−1^)	200–987	4–7000	66–71
TA^a^ (mg per L CaCO_3_)	83–200	128	302–372
Phosphate (mg L^−1^)	4–27.6	3.43	10.8–167
COD (mg L^−1^)	375–4155	80–212000	477–876
BOD5 (mg L^−1^)	48–1200	218–9810	44–50
TH^a^ (mg per L CaCO_3_)	—	44	53–68

a EC: electrical conductivity, TA: alkalinity rate, TH: hydrotimetric rate (water hardness).

Due to its low level of pollutants concentration, laundry greywater has a high potential for recycling and can be reused as service water for irrigation, toilet flushing or even in washing activities.^23^ However, microbe proliferation and the spread of infection must be considered when reusing laundry greywater, especially in cases of bad levels of maintenance for water storage systems.^22^Table 3 shows the recycled water quality standards based on the protection of human health and the environment and suitability for reuse in applications such as laundry, toilet flushing and irrigation.

**Table 3:** Water recycling standards and suitability for the intended reuse application adapted from ref. 19, 24 and 25

Parameters	Laundry water	Toilet flushing	Irrigation
pH	6.0–9.0	6.0–9.0	6.0–8.5
Turbidity (NTU)	2	2	No limits
TDS (mg L^−1^)	2000	1000	No limits
TSS (mg L^−1^)	10	30	15–35
TH (mg per L CaCO_3_)	90	No limits	No limits
BOD5 (mg L^−1^)	10	10–30	15–30
Fecal coliform (CFU/100 mL)	75% not detected^a^, 25 max	200 average^b^, 800 max	200 average^b^, 800 max

a Not detected in 75% of samples.

b 200 CFU/100 mL average for year.

### 2.2. Laundry wastewater micropollutants

The selection of which technology or method to use in recycling laundry wastewater depends on the properties and the type of the associated micropollutants.^26^ For instance, using a membrane bioreactor (MBR) technology in recycling laundry wastewater can remove up to 99.9% of microplastic pollutants. However, using UV irradiation treatment can only cause a microplastic mass loss of about 23.6%.^27,28^ In contrast, using UV irradiation in combination with other oxidation treatments, such as ozonation (O_3_) and hydrogen peroxide (H_2_O_2_), can be more effective in removing pharmaceutical antibiotics from laundry wastewater.^29^ Moreover, using bioremediation, which involves a combination of algae and bacteria, can be highly effective in treating nitrogen compounds that are byproducts of washing detergents. Meanwhile, adsorption techniques are suitable for the removal of phosphates and synthetic dyes.^30,31^ Biofilter and photo-Fenton represent promising technologies for treating anionic and non-ionic surfactants, while microfiltration excels at removing cationic surfactants.^32,33^ Furthermore, electrocoagulation applications have demonstrated enhanced efficacy in addressing synthetic dyes, pharmaceuticals, and organic compounds.^34–36^Table 4 summarises the impact of several wastewater treatment technologies on common laundry wastewater micropollutants.

**Table 4:** The impact of wastewater treatment technologies on laundry wastewater micropollutants^a^

Micropollutants	Study	Treatment	Conditions	Treatment impact
Microplastic/microfibers: (size 1 μm–5 mm)	Talvitie *et al.*^37^	Membrane bioreactor (MBR)	Membrane pore size = 0.4 μm, effective membrane area = 8 m^2^, HRT* from 20 to 100 h and flow rate between 40 and 90 l h^−1^	Removal efficiency of MPs is 99.9%
	Yaranal *et al.*^36^	Electrocoagulation (aluminum sheets electrodes)	Electrodes surface area = 6.42 × 10^−3^ m^2^, operating time = 25 min and current density = 300 A m^−2^	Removal efficiency of MPs is 97.9%
	Napper *et al.*^28^	External filters	Mesh pore size = 60 μm	Removal efficiency of MPs is 78%
	Easton *et al.*^27^	(a) UVC/H_2_O_2_*	H_2_O_2_ dose = 500 mg l^−1^, UVC irradiation = 4.0 mW cm^−2^ operating time = 48 h	(a) Mass loss of MPs is 52.7%
		(b) UVC treatment only		(b) Mass loss of MPs is 23.6%
	Mais *et al.*^38^	Electrolysis (mixed metal oxide anodes)	Current density = 20 mA cm^−2^	Weight loss of MPs is 70%
Nutrients: phosphates nitrogen (N) compounds	Adesoye *et al.*^39^	Chemical coagulants (alum and ferrous sulphate)	pH = 10.20 Al_2_(SO_4_)_3_·18H_2_O (alum) = 0.0025 g FeSO_4_·7H_2_O (ferrous sulphate) = 0.0013 g	Phosphate removal 30%
	Agustina *et al.*^30^	(a) Adsorption (active carbon – AC)	Adsorbent height = 40 cm initial phosphate concentration = 2 mg l^−1^ AC specific surface area = 26 m^2^ g^−1^ NZ specific surface area = 48 m^2^ g^−1^	(a) Phosphate removal 60%
		(b) Adsorption (natural zeolite – NZ)		(b) Phosphate removal 90%
	Titah & Nasir^40^	Adsorption (granular activated carbon + zeolite)	Adsorbent composition ration = 50 : 50, adsorbent mass = 12 g contact time = 150 minutes	Phosphate removal 57.14%
	Rodzi *et al.*^31^	Bioremediation (algae and bacteria mixture)	Initial ammonia-N = 1.29 mg l^−1^, nitrate = 0.119 mg l^−1^ and nitrite = 0.001 mg l^−1^ mixture mass = 0.5 g for ammonia–nitrogen, 2 g for nitrate-N and 1 g for nitrite-N	Removal efficiency of ammonia-N is 71%, nitrate-N is 99% and nitrite-N is 96%
Anionic surfactants: LAS* MBAS* SDBS*	Melián *et al.*^32^	(a) Photo-fenton	(a) Initial LAS = 9.42 ± 0.85 mg l^−1^ pH = 2.8, Fe(iii) = 33.5–500 mg l^−1^ and H_2_O_2_ = 408–4080 mg l^−1^	(a) LAS removal by 82.4%
		(b) Biofilter (BF)	(b) pH = 7 and max HRT* = 52 h	(b) LAS removal by 98%
	Yaranal *et al.*^36^	Electrocoagulation (aluminum sheets electrodes)	Electrodes surface area = 6.42 × 10^−3^ m^2^, operating time = 25 min and current density = 300 A m^−2^	MBAS removal by 91.2%
	Collivignarelli *et al.*^41^	(a) Thermophilic aerobic membrane reactor (TAMR)	TSS = 150–190 kg TSS per m^3^, SLR* = 0.0133–0.033 kg COD per kg TSS per day pH = 6.5–7.5 and O_2_ = 1.5 mg l^−1^ NF operating pressure = 20–30 bar, NF contact angle = 28.77 ± 2.43°, AC contact time = 15–20 min operating temp. TAMR = 48–50 °C, NF = 35–40 °C and AC = 25–30 °C	(a) MBAS removal by 49.5 ± 2.8%
		(b) Nano filtration (NF) + adsorption (active carbon)		(b) MBAS removal by 52.5 ± 2.8%
		(c) TAMR* + NF*		(c) MBAS removal by 69.8%
		(d) TAMR + NF + AC*		(d) MBAS removal by 76.7%
	Ncibi *et al.*^42^	Adsorption (multi-walled carbon nanotubes)	Average MWCNT* length = 5 μm, purity >95%, temperature = 25 °C, adsorbent dosage = 0.1 g l^−1^, pH = 2, surfactant concentration = 10–150 mg l^−1^, sonication time = 0.5 h	SDBS removal capacity is 312 mg g^−1^
Non-ionic surfactants: BiAS* NPEOs* TAS* TX-100*	Melián *et al.*^32^	(a) Photo-fenton	(a) Initial BiAS = 37.36 ± 0.17 mg l^−1^ pH = 2.8, Fe(iii) = 33.5–500 mg l^−1^ and H_2_O_2_ = 408–4080 mg l^−1^	(a) BiAS removal 98.7%
		(b) Biofilter (BF)	(b) pH = 7 and max HRT* = 52 h	(b) BiAS removal 98.7%
	Khajvand *et al.*^43^	Adsorption (AC)	Initial NPEOs = 971.3 ± 93.5 μg l^−1^, adsorbent particle size = 0.25–1 mm, temperature = 20 ± 2 °C, pH = 7.0 ± 0.5, shaker speed = 150 rpm, and treatment time = 180 min	NPEOs removal >99%
	Collivignarelli *et al.*^41^	(a) Nano filtration (NF)	NF operating pressure = 20–30 bar, NF contact angle = 28.77 ± 2.43° AC contact time = 15–20 min SLR* = 0.0133–0.033 kg COD per kg TSS per day, TSS = 150–190 kg TSS per m^3^, pH = 6.5–7.5 and O_2_ = 1.5 mg l^−1^ operating temp. TAMR = 48–50 °C, NF = 35–40 °C and AC = 25–30 °C	(a) TAS removal 74.1 ± 2.0%
		(b) Nano filtration (NF) + adsorption (activeCarbon)		(b) TAS removal 91.4 ± 1.2%
		c. TAMR + NF + adsorption (AC)		(c) TAS removal 95.3 ± 0.8%
	Ncibi *et al.*^42^	Adsorption (multi-walled carbon nanotubes)	Average MWCNT* length = 5 μm, purity >95%, temperature = 25 °C, adsorbent dosage = 0.1 g l^−1^, pH = 6, surfactant concentration = 10–150 mg l^−1^ and sonication time = 0.5 h	TX-100 removal capacity 359 mg g^−1^
Cationic surfactants: TEAQ* CTAB*	Klimonda & Kowalska^33^	Micro filtration (MF) (0.14 μm pore size)	Initial TEAQ concentration = 1000 mg l^−1^, processes time = 120-min cycles, transmembrane pressure = 0.3 MPa, and flow velocity = 5 m s^−1^	TEAQ removal by 99%
	Ncibi *et al.*^42^	Adsorption (multi-walled carbon nanotubes)	Average MWCNT* length = 5 μm, purity >95%, temperature = 25 °C, adsorbent dosage = 0.1 g l^−1^, pH = 8, surfactant concentration = 10–150 mg l^−1^ and sonication time = 0.5 h	CTAB removal capacity 156 mg g^−1^
Synthetic dyes: direct red 81 reactive black-5 methylene blue (MB) methyl orange (MO)	Aoudj *et al.*^34^	Electrocoagulation (EC) – aluminum electrodes	Current density = 1.875 mA cm^−2^, initial pH = 6, inter-electrode distance = 1.5 cm	Direct red 81 removal by 98%
	Kumar *et al.*^44^	Ultra-filtration (polysulfone hollow fibre membrane)	Membrane pore size = 1 nm, diameter = 1 mm	Reactive Black-5 removal >99%
	Rahman^45^	Adsorption activated carbon (AC)	Adsorbent dosage = 0.25 g/50 mL, initial dye concentration = 10.0 mg l^−1^, pH = 10, temperature = 45 °C, mixing rate = 175 rpm, and mixing time = 2.0 h	Methylene blue (MB) removal by 97.18%
	Ahmed *et al.*^46^	Chemical coagulation (FeCl_3_/lime)	Coagulant dosage for MO = 200 mg/70 l coagulant dosage for MB = 70 mg/100 l pH = 6.0–8.0	Removal efficiency of methyl orange (MO) is 97.78% and methylene blue (MB) is 95.54%
Pharmaceutical: antibiotics antidepressants analgesics lipid regulator lipid regulator X-ray contrast media disinfectants	Balarak *et al.*^35^	Electrocoagulation (EC) – aluminum electrodes	Voltage = 60 V, pH = 7, electrolysis time = 75 min potassium chloride (KCL) concentration = 3 g l^−1^	Amoxicillin removal 98.8%
	Moarefian *et al.*^47^	Nano filtration (NF)	Operating temperature = 298 K (24.85 °C), pressure of 2 MPa, pH = 9.0 and initial amoxicillin concentration = 20 ppm	Amoxicillin removal 99.09%
	Shaykhi Mehrabadi^29^	UV/O_3_/H_2_O_2_	H_2_O_2_ concentration = 0–20 millimolar (mM), initial pH = 3–11	Amoxicillin removal 100%
	Dogan & Kidak^48^	UV and UV/H_2_O_2_	UV fluence rate = 2.3 W m^−2^, H_2_O_2_ concentration = 588 mg l^−1^, plug flow reactor radius = 1.75 cm and peristaltic pump speed = 10 rpm	Amoxicillin removal 55% using UV and 90% using UV/H_2_O_2_
	Khan *et al.*^49^	Adsorption (packed activate carbon)	—	Removal efficiency of antibiotics is 70–90%, antidepressants is 70–90%, analgesics >90% lipid regulator is 70–90%, X-ray contrast media is 70–90%, and disinfectants is >90%
Organic matter	Yaranal *et al.*^36^	Electrocoagulation (aluminum sheets electrodes)	Electrodes surface area = 6.42 × 10^−3^ m^2^, operating time = 25 min and current density = 300 A m^−2^	COD removal 86.3%
	Titah & Nasir^40^	Adsorption (granular activated carbon + zeolite)	Adsorbent composition ration = 50 : 50, adsorbent mass = 12 g contact time = 150 minutes	COD removal 63.11%
	Rodzi *et al.*^31^	Bioremediation (algae and bacteria mixture)	Initial COD = 216 mg l^−1^, mixture concentration = 2 g processing time = 7 days	COD removal 61%
	Cüce & Aydın Temel^50^	Conventional fenton (CF) and Photo-Fenton (PF)	pH = 3, H_2_O_2_/Fe^2+^ ratio for CF is 900/400 and 600/400 for PF	COD removal efficiency of 90% for CF and 99% for PF
	Solehudin *et al.*^51^	Aerobic bioreactor (a) suspended growth, and (b) attached growth	Temperature = 26–28 °C operating time = one week (a) suspended growth system: pH = 6.3–7.1 and influent COD = 377.5 mg L^−1^ (b) attached growth system: pH = 6.4–7.4, influent COD = 755.5 mg l^−1^	COD removal efficiency of 78% for (a) suspended growth system and 94% for (b) attached growth system

a Hydraulic Retention Time (HRT), Microfilter (MF), UV irradiation and hydrogen peroxide treatment (UVC/H_2_O_2_), linear alkylbenzene sulfonate (LAS), Methylene Blue Active Substances (MBAS), Thermophilic Aerobic Membrane Reactor (TAMR), Sludge Loading Rate(SLR), Nanofiltration (NF), Activated Carbon (AC), Multi-Walled Carbon Nano-Tubes (MWCNTs), Bismuth Active Substance (BiAS), cationic surfactant TEAQ (dihydrogenated tallowethyl hydroxyethylmonium methosulfate & ditallowethyl hydroxyethylmonium methosulfate), critical micelle concentration (CMC), Sodium Dodecyl Benzene Sulfonate (SDBS), Cetyltrimethylammonium Bromide (CTAB), Octylphenolethoxylate (TX-100), Nonylphenol Ethoxylates (NPEOs).

### 2.3. Microplastics (MPs)

Microplastics (MPs) encompass solid polymeric particles or matrices with sizes ranging from 1 μm to 5 mm, existing in various shapes and types and can have adverse effects on ecosystems. Synthetic microfibers (MFs) represent the most commonly detected types of MPs in the environment, generally originating from textile industries, homes and commercial laundries. Polyester polyethylene terephthalate (PET) has been identified as the predominant material among microfibers found in effluent from commercial washing machines.^52,53^ According to Dreillard *et al.*,^54^ the average diameters of microfibers (MFs) generated by washing textiles range from 8 to 17 μm, with 40 to 75% of them having lengths between 50 and 200 μm. Current research efforts focus on developing environmentally friendly, renewable and cost-effective separation media and innovative systems for treating microplastics in laundry wastewater. For instance, Bhuyan *et al.*^55^ succeeded in separating around 99% of polyethene microspheres (53–63 μm) from water utilising superhydrophobic geopolymer foam as a filtration medium and achieved a removal efficiency of about 84% for microplastics (approximately 2 μm–2 mm) when treating laundry washing effluents. Additionally, Liu *et al.*^56^ removed around 96% of microplastic particles with average size of 25 μm using a delignified wooden filter coated with cellulose nanofiber. Furthermore, Jiang *et al.*^57^ attained 100% removal efficiency of suspended solids with a cut-off size of approximately 10 nm using nanocellulose hydrogel film in a filtration system. Biological treatments are often regarded as the most ecologically safe. However, the biodegradability of microplastics can be affected by several factors such as particle morphology, the chemical composition of MPs, and process conditions. Therefore, employing an economical and efficient pretreatment before biodegradation can ensure a more thorough remediation of microplastics.^58^ For instance, Easton *et al.*^27^ observed a formation of shallow holes, pits and cracks across the polyester fibre surface when using UV/H_2_O_2_ oxidation process. Such a change in the surface of MPs facilitates the processes of microbial adhesion and biofilm formation during bioremediation.

## 3. Laundry wastewater treatment technologies

**Fig. 2:**
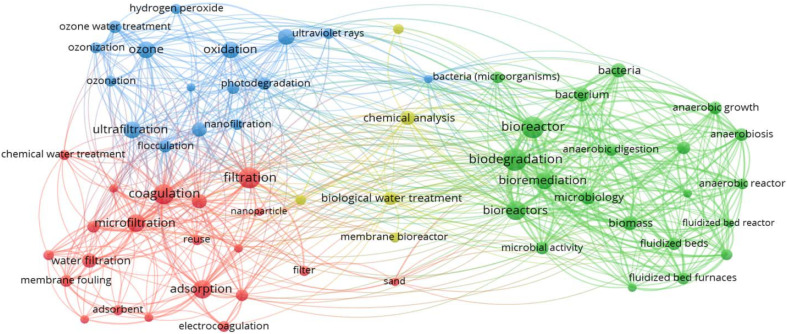
Bibliometric analysis results of laundry wastewater treatment techniques.^60^

**Fig. 3:**
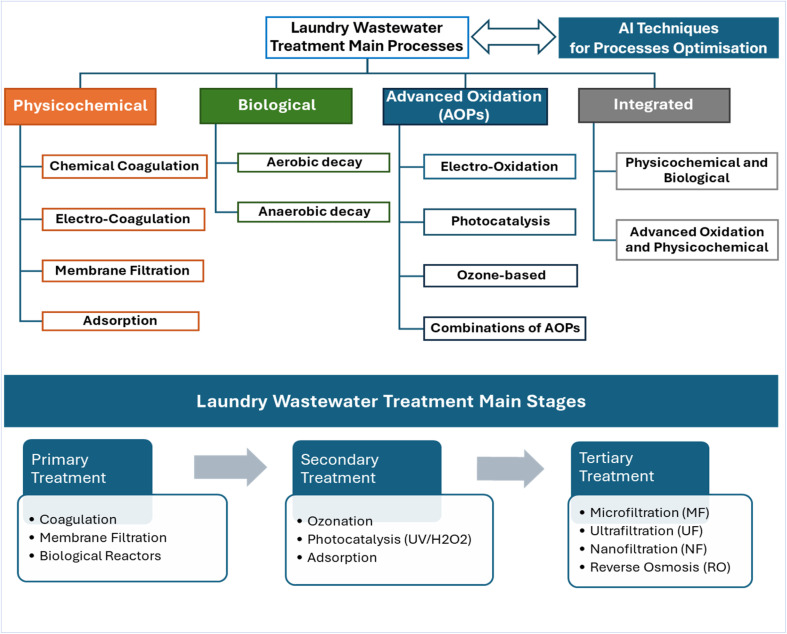
Laundry wastewater treatment main processes and stages.

Studies that employ simple physical treatments and disinfection techniques for greywater recycling were reported in the early 1970s, in which coarse filters and membranes were utilised. In the following decade, biological treatment technologies such as aerated bioreactors and rotating biological contactors were investigated. Later, advanced technologies such as membrane bioreactors (MBRs), photocatalysis and chemical treatments including conventional and electrocoagulation were developed.^59^ According to Kumar *et al.*,^21^ laundry greywater treatment technologies can be classified into three main branches physicochemical, biological and a combination of both techniques. Fig. 2 shows a bibliometric analysis conducted on the literature review of various laundry greywater treatment technologies for the period from 1900 to 2020.^60^ These technologies were classified into the coagulation, adsorption and filtration, biological treatment, and oxidation processes groups. Additionally, results show that the keywords most cited in treatment techniques, with more than 50 citations, are filtration, reverse osmosis, coagulation, electrocoagulation, adsorption, biomass, and UV radiation. Furthermore, these wastewater treatment techniques are often applied sequentially to remove the different micropollutants. Artificial intelligence (AI) has emerged as a powerful tool for the management and optimisation of wastewater treatment systems. Fig. 3 summarises laundry wastewater treatment main processes and illustrates the classification of the primary processes utilised in each treatment stage.

### 3.1. Chemical coagulation

**Fig. 4:**
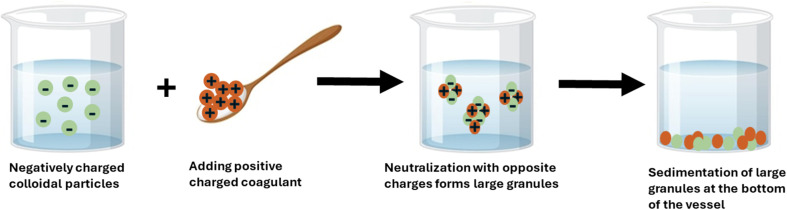
Schematic diagram of the chemical coagulation mechanism.

Coagulation is a chemical process in which a coagulant is added to contaminated water to help aggregate fine colloidal particles dispersed in water into larger clumps. Coagulants are often used to reduce turbidity, suspended solid, organic matter and chemical oxygen demand (COD) in the wastewater. These coagulants can be categorised into two main groups: natural and synthetic. Natural coagulants have various organic origins such as animals, plants, and microorganisms. In contrast, the most used synthetic coagulant in water treatment are aluminium and iron salts in addition to organic polymers. Examples of inorganic coagulants include aluminum sulfate Al_2_(SO_4_)_3_, ferric sulfate Fe_2_(SO_4_)_3_ and ferric chloride (FeCl_3_). As shown in Fig. 4, colloidal particles suspended in water with similar electrical charges (negatively charged) tend to repel each other, leading to increased water turbidity; the addition of coagulants (positively charged) helps neutralise these electrical charges and encourages suspended particles to stick together to form larger granules and thus ease the separation process through sedimentation.^61,62^

### 3.2. Electro-coagulation (EC)

**Fig. 5:**
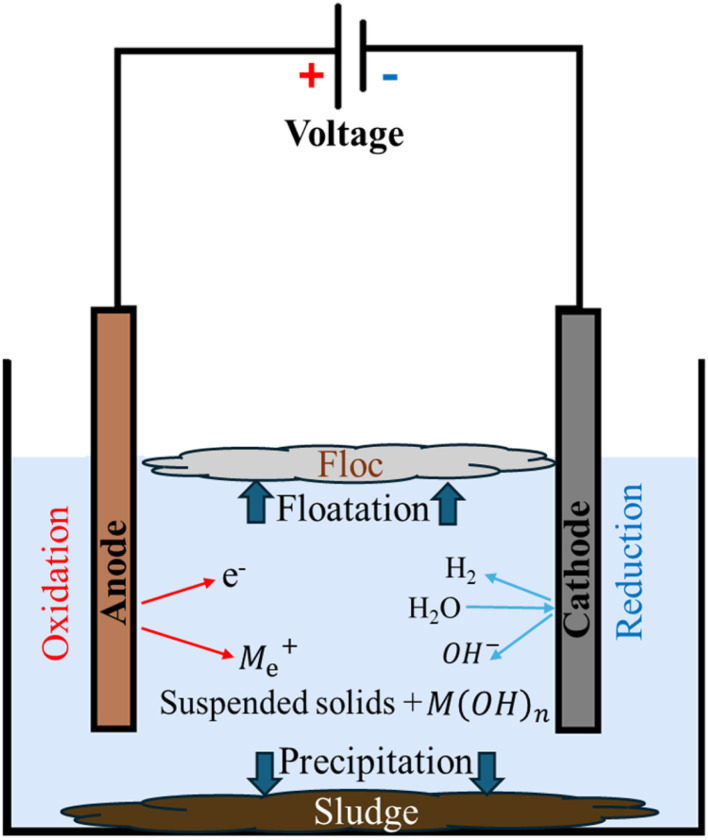
Treatment mechanism of electrocoagulation process.

Electrocoagulation is an electrochemical wastewater treatment that consists of three consecutive stages: coagulant formation through the oxidation of metal electrodes, destabilisation of pollutant particles charge, and aggregation of these destabilised particles to form clumps. As shown in Fig. 5, when wastewater passes through an electrocoagulation cell, the anode produces dissolved metal ions M_e_^+^ such as iron Al^3+^ and Fe^2+^, see eqn (1). These metal ions neutralise the negative surface charge of the wastewater's suspended particles and help them form larger flocs. Additionally, the cathode electrode decomposes the water in a reduction reaction into hydroxyl ion and hydrogen gas, see eqn (2). Furthermore, the metal hydroxide can be formed as a result of the reaction between the produced metal ions M_e_^+^ and the hydroxide ions OH^−^, see eqn (3).^21,63^

Eqn (1)–(3) describe the reactions in the electrocoagulation cell:^63^

1 M_(s)_ → M_aq_^*n*+^ + *n*e^−^

2 2H_2_O + 2e^−^ → 2OH^−^ + H_2_

3 M_e_^*n*+^ + *n*OH^−^ → M(OH)_*n*(s)_

where M_(s)_ is solid metal, M_aq_^*n*+^ is dissolved metal ion, M(OH)_*n*(s)_ is metal hydroxide.

These metal hydroxide M(OH)_*n*_ can help in removing heavy metal and suspended solids through complexation and co-precipitation.^21,63^ For instance, Amorphous Al(OH)_3(s)_ (sweep flocs), upon formation, possess large surface areas that facilitate the rapid adsorption and entrapment of pollutants. The adsorption of heavy metal ions by metal hydroxide flocs involves a series of complex processes, including complexation, sweep coagulation, co-precipitation, and electrical neutralization. Of these mechanisms, the complexation reaction is the most significant, as outlined in eqn (4):^64^

4 *n* ≡ Al − OH + M_e_^*n*+^ = (≡Al − O)_*n*_ → M_e_ + *n*H^+^

where ≡ is the surface of the particle, *n* ≡ Al − OH is surface hydroxyl groups on aluminium-based material, M_e_^*n*+^ is a heavy metal ion with charge *n*+, → is coordinate bonds, *n*H^+^is hydrogen ions.

There are many factors affecting electrocoagulation efficiency including the pH of the medium, electrical current density, the type of electrode material, the distance between the electrodes and the suspended solids concentration in the wastewater which can decrease the effective surface area of the electrodes.^65^

### 3.3. Membrane filtration

**Fig. 6:**
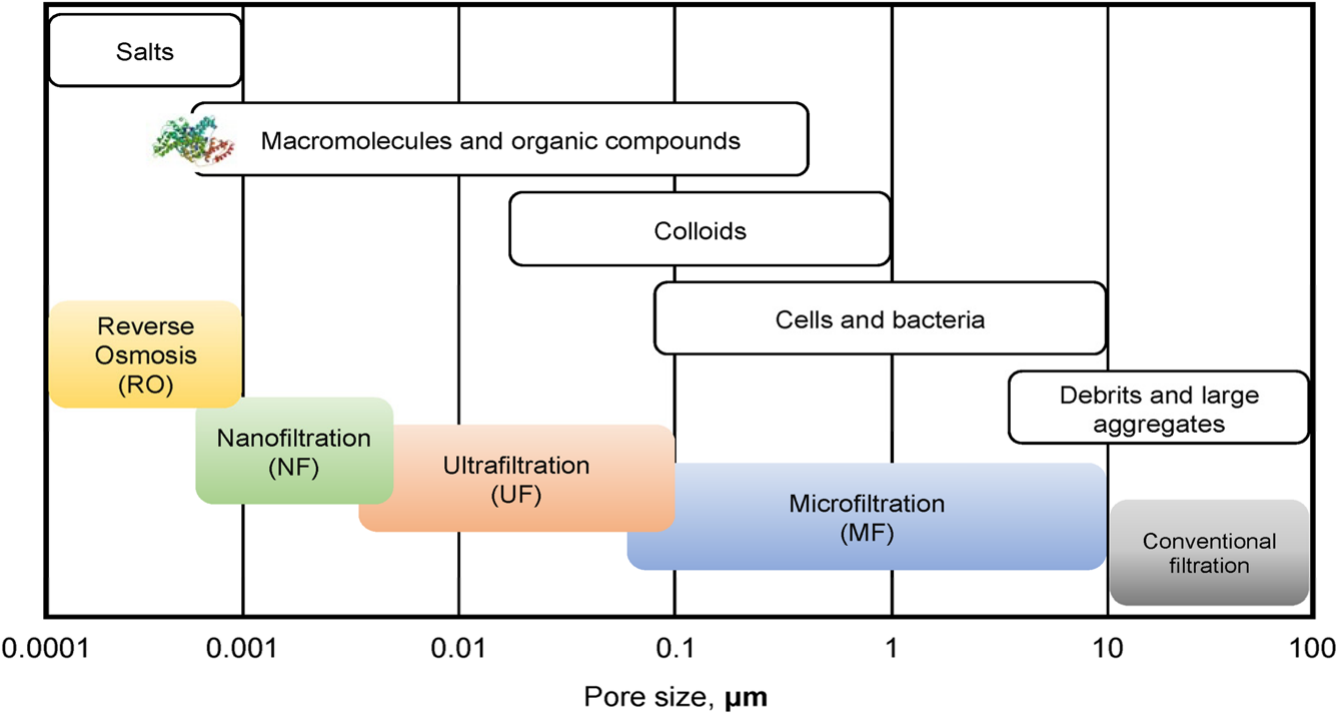
Types of membrane processes and dimensions of various common pollutants.^69^

Membrane filtration is widely used in wastewater treatment because it is an economical, environmentally safe system and often has high separation efficiency.^66,67^ The selection of an appropriate membrane for a filtration process depends on the volume of the various compounds in the wastewater. Membrane filtration processes are divided into several types based on their average pore sizes including conventional filtration, microfiltration (MF), ultrafiltration (UF), nanofiltration (NF) and reverse osmosis (RO).^68^ Thus, each filtration system can effectively block or reduce one of the fouling species, as shown in Fig. 6. For instance, UF and MF membranes are suitable for removing biological matter such as cells and bacteria, while RO and NF processes are better options for salts and ion removal.^69,70^

**Fig. 7:**
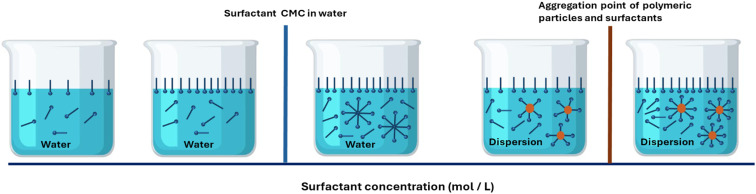
Critical micelle formation concentration in polymeric particles dispersion.

The concentration of surfactants in the laundry greywater is a key factor in choosing a suitable membrane for the filtration process.^71,72^ When the critical micelle concentration (CMC) is reached in an aqueous solution, surfactants aggregate to form spherical micelles within that solution.^73^ However, the value of CMC in the solution changes with the change in the concentration/volume and type of pollutants such as polymeric particles and other suspended solids, see Fig. 7.^74^ The sizes of these spherical micelles can range from 2 to 20 nm. Thus, microfiltration (MF) and ultrafiltration (UF) can be utilised to separate aggregated surfactants when CMC is attained in the solution. Meanwhile, nanofiltration is used in situations where CMC is not reached, and organic compounds are present.

The limitation of traditional membrane separation processes lies in the accumulation of impurities such as proteins and microorganisms, leading to the obstruction of both the membrane's surface and its pores, consequently resulting in membrane fouling.^75^

### 3.4. Adsorption

**Fig. 8:**
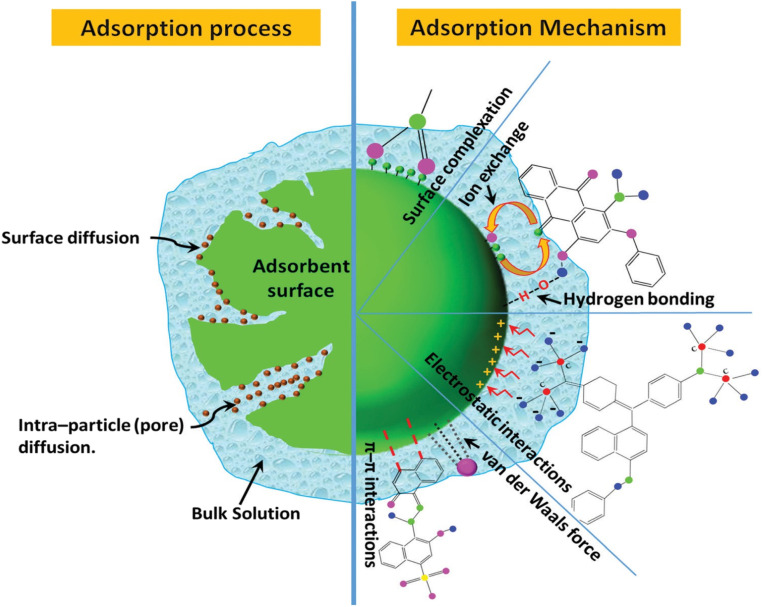
Adsorption phenomenon process and mechanism.^77^

The adsorption phenomenon occurs when a solution comprising absorbable solutes contacts a solid material with high porosity, where liquid–solid intermolecular forces cause some of the solutes to diffuse into the surface of the solid material (absorbent surface).^76^ Furthermore, the adsorption mechanisms of water pollutants on an adsorbent surface involve electrostatic attraction, van der Waals forces, π–π interactions, hydrogen bonding, hydrophobic interaction, and acid–base reactions; see Fig. 8.^77^ For instance, the adsorption of anionic solutions such as dyes can be attained by ionic exchange and electrostatic attraction or through surface complexation mechanism, which describes the binding of solution ions to functional groups in the absorbent surface in addition to the electrostatic attraction between their surfaces.^78^

Adsorbents can be divided into natural and synthetic adsorbents. Natural adsorbents such as charcoal, clay, and zeolites are relatively cheap and have abundant supplies. Whereas, synthetic adsorbents are generated from agricultural products, sewage sludge, and industrial waste in addition to the polymeric adsorbents.^76,79^ According to Rodrigues,^80^ the suitability of an adsorbent to any treatment process depends on its surface area, pore volume, adsorption capacity, low cost, availability, ease of regeneration and modification, physical and chemical characteristic stability, and its potential to provide fast mobility. Thus, the popularity of iron oxide nanoparticles (IONPs) as adsorbents has increased due to their high surface area and the ability to adjust the particles' surface to target specific pollutants in addition to the ease of using magnetic-based separation techniques to retrieve them.^81–85^ Furthermore, graphene-based nanomaterials such as graphene oxide (GO) are highly effective for decontaminating dyes and antibiotics. In contrast, nanoparticles of silver, iron and copper are preferred for the removal of pathogens.^86,87^

### 3.5. Biological treatments

Anaerobic and aerobic treatment processes are extensively employed for the elimination of organic pollutants in wastewater, as assessed by parameters such as biochemical oxygen demand (BOD) and chemical oxygen demand (COD).^88^ This method entails the controlled utilisation of microorganisms, specifically bacteria, to metabolise organic substances in wastewater as their nutrient source. Aerobic microorganisms use oxygen to biodegrade organic waste, converting it into biomass and carbon dioxide (CO_2_).^89^ While anaerobic microorganisms break down complex organic matter into CO_2_, H_2_O and CH_4_ through a series of reactions that include hydrolysis, acidogenesis, acetogenesis and methanogenesis. This proved to be an effective approach for the removal of specific organic materials from wastewater.^90,91^ Due to the involvement of living organisms, careful consideration must be given to factors influencing the growth and health of the microbial culture. This includes ensuring an adequate supply of organic materials as food, the availability of essential nutrients such as phosphorus and nitrogen, maintaining a suitable temperature range, and providing a non-toxic and relatively stable environment devoid of temperature shocks and similar disturbances.^89,92^

Biological reactors can utilise both anaerobic and aerobic treatment systems. Anaerobic bioreactors encompass membrane bioreactor (MBR), fluidized bed reactors, up-flow anaerobic sludge blanket (UASB), granular sludge bed and up-flow filter bioreactor. These bioreactors offer advantages such as low biomass deposition, recirculation capability, reduced energy consumption, minimal sludge production, and the ability to separately control hydraulic retention time (HRT) and sludge retention time (SRT). Aerobic reactors can be categorised according to the microbial growth state, specifically as attached or suspended growth systems. Attached growth systems encompass submerged systems such as up-flow or downflow packed beds, non-submerged systems that include trickling filters, and suspended packing for the attached growth process, which includes biofilm reactors. Suspended growth systems involve activated sludge systems that can be managed using various configurations such as continuously stirred tank (CSTR), plug-flow, sequencing batch (SBR) and jet loop reactors.^89,92–94^ In a study to test the efficiency of moving bed bioreactor (MBBR), Bering *et al.*^95^ used two stages of MBBR under aerobic conditions. The study demonstrated a substantial reduction, with BOD decreasing by 95–98%, COD by 89–94%, and the combined levels of nonionic and anionic surfactants by 85–96%. Munawar *et al.*^96^ reported a reduction in MBAS by 27–76%, TSS by 80–90%, phosphate by 21–72%, COD by 62–90% and BOD by 56–82% through the utilisation of a downflow hanging sponge (DHS) aerobic bioreactor as a laundry wastewater treatment unit. The study of Najmi *et al.*^97^ focused on treating personal care products (PCP) greywater using a submerged membrane bioreactor (SMBR) system. This SMBR system succeeded in removing up to 98.20%, 99.96% and 99.97% of the organic antimicrobial agent triclosan (C_12_H_7_C_l3_O_2_), the preservatives methylparaben and propylparaben, respectively. By employing an oxygen-based membrane biofilm reactor for greywater treatment, Zhou *et al.*^98^ achieved notable results, including a 98% reduction in LAS, a 99% decrease in inorganic nitrogen levels and a 95% reduction in total COD.

### 3.6. Advanced oxidation

Advanced oxidative processes (AOPs) have been established as a highly promising approach for eliminating pollutants that are typically resistant to removal through conventional treatment methods. AOPs rely on the production of extremely reactive radical oxidative species (ROS), including the hydroxyl radical (OH˙) and other related entities such as the hydrogen radical (H˙), hydrated electron (e_aq_^−^), singlet oxygen (^1^O_2_), hydroperoxyl radicals 
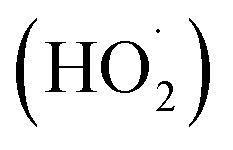
, superoxide radicals (O_2_˙^−^). Advanced oxidative processes include ozone-based AOP, the Fenton process, electrochemical oxidation, photolysis, photocatalysis, sonolysis, and a combination of these methods with conventional techniques.^99–101^ These processes exhibit significant efficacy in eliminating non-biodegradable contaminants and can function as sophisticated approaches for treating wastewater. However, challenges associated with high level of energy consumption, chemical inputs, catalyst support corrosion, suboptimal light utilisation, constrained ozone mass transfer, and operational expenses frequently limit the widespread application of these processes in wastewater treatment. Furthermore, the diverse origins and characteristics of laundry greywater can influence the effectiveness and procedural nuances of different AOPs. For instance, electro-Fenton faces significant challenges, particularly pertaining to the costs and lifespan of electrodes, as well as the elevated consumption of electrical energy. Similar issues, such as the demand for acidic pH conditions and the formation of sludge inherent in the Fenton and photo-Fenton processes, are also evident in electro-Fenton. These processes prove unsuitable for the treatment of laundry wastewater, given its alkaline nature and the considerable pH adjustments that would be required.^100,102^ Moreover, the primary challenges associated with photolytic and photocatalytic methods stem from the diminished UV-transmittance resulting from elevated turbidity levels. While sonochemical oxidation techniques are notably energy-intensive process that is impractical for the treatment of large volumes of wastewater, making it more suitable as a supplementary method to assist other AOPs.^100,103^

#### 3.6.1 Electro-oxidation (EO)

Electrooxidation is an electrochemical process often used in wastewater treatment to reduce the load level of COD, degrade the organic pollutants, antibiotics and treat water color. The electrooxidation process involves two types of reactions: direct and indirect oxidation reactions.^104,105^ Direct oxidation reaction happens when water is adsorbed to the surface of the anode and oxidised causing the formation of hydroxyl radicals OH˙, see eqn (5). Subsequently, these hydroxyl radicals oxidise the organic pollutants R into biodegradable compounds RO, see eqn (6). Furthermore, hydroxyl radicals can completely degrade the organic pollutants into water and carbon dioxide, as shown in eqn (7).^63,106^

5 H_2_O + M → M(OH˙) + H^+^ + e^−^

6 R + M(OH˙) → M + RO + H^+^ + e^−^

7 R + M(OH˙) → M + *m*CO_2_ + *n*H_2_O + H^+^ + e^−^

where M represents the active site of the anode surface, and R represents the organic pollutant.

The indirect oxidation process arises due to the electrolysis of water in both the anode and cathode electrodes. Where organic pollutants are degraded by reactive compounds resulting from the electrooxidation of other inorganic chemical compounds in the wastewater. These reactive compounds can include hydrogen peroxide H_2_O_2_, hypochlorous acid HClO, peroxydisulfuric acid H_2_S_2_O_8_ and ozone O_3_, as shown in eqn (8)–(10).^63,106^

8 O_2_ + 2H^+^ + 2e^−^ → H_2_O_2_

9 2SO_4_^2−^ + 2H^+^ → H_2_S_2_O_8_ + 2e^−^

10 Cl^−^ + 2H_2_O → HClO + H_3_O^+^ + 2e^−^

#### 3.6.2 Photocatalysis

**Fig. 9:**
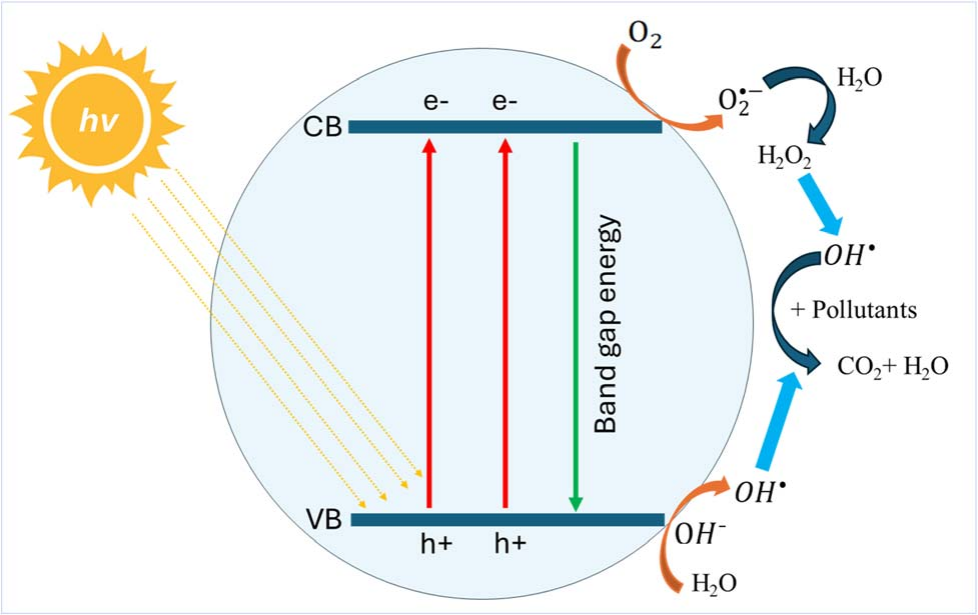
Scheme of the photocatalytic degradation mechanism.

Photocatalysts are materials that enhance the speed of chemical reactions when exposed to irradiation, typically by a light source. When irradiated, the photocatalyst interacts with oxygen, water, and hydroxyl groups, leading to the production of OS such as hydroxyl radicals (OH˙) and superoxide radical anions (O_2_˙^−^), all of which are potent oxidising agents. As one of the AOPs, the utilisation of photocatalysis has experienced significant growth due to its reputation as an environmentally friendly, sustainable, and energy-efficient technology suitable for addressing non-biodegradable, complex, and highly concentrated pollutants in wastewater.^107^ The photocatalyst captures energy from UV, visible, or solar irradiation sources within the water to generate powerful oxidising agents capable of breaking down enduring organic pollutants in the water, resulting in the production of carbon dioxide (CO_2_) and water (H_2_O).^108^ As shown in Fig. 9, when subjected to irradiation, the energy of incident photons (*hv*) activates the photocatalyst, causing the transfer of electrons from the valence band (VB) to the conduction band (CB). In the conduction band, electrons are released and combined with oxygen to form superoxide radical anions (O_2_˙^−^). Simultaneously, the surface of the photocatalyst in the valence band becomes positively charged and accepts electrons from water, leading to the generation of hydroxyl radicals (OH˙).^109,110^

Photocatalysis can be categorised into two classes: homogeneous and heterogeneous. This classification is determined by the physical state in which the reactants are present. Homogeneous photocatalysis involves the photocatalyst and reactants being in the same phase. In homogeneous photocatalysis, free radicals are generated by exposing light to the uniform molecules of oxidising agents such as ozone (O_3_) and hydrogen peroxide (H_2_O_2_). Some examples of homogeneous photocatalysis include ozonation, UV/H_2_O_2_, and UV/H_2_O_2_/O_3_. In contrast, in heterogeneous photocatalysis, the photocatalysts are entirely distinct from the reactants, typically dispersed within them. Heterogeneous photocatalysts are classified based on their structural composition and material arrangement, which influence their efficiency and functionality. The simplest type, powder photocatalysts, consists of a single material that can be undoped or doped for enhanced properties.^84^ Bimetallic photocatalysts incorporate two metals to improve charge separation and catalytic activity.^111^ Decorated photocatalysts feature core nanoparticles covered with smaller particles, increasing surface interactions.^112^ Core–shell structures involve a core nanoparticle enclosed by another material, enhancing stability and charge dynamics.^113^ Composite photocatalysts embed core nanoparticles onto a larger surface, improving dispersion and charge transfer for enhanced performance.^114^ These photocatalysts frequently consist of semiconductor metal oxides, selected for their inherent capability to absorb light such as titanium dioxide (TiO_2_) and zinc oxide (ZnO). This absorption process facilitates electron transfer, culminating in the generation of ROS.^110^ The ongoing endeavours in exploring photocatalysis for wastewater treatment continue to evolve, incorporating strategies to enhance the spectral response within the visible region of photocatalytic oxides to facilitate the use of solar radiation as an activation source.^115,116^ Doping TiO_2_ with metallic ions is a popular strategy for improving its photocatalytic efficiency. As an illustration, the utilisation of T-DK (1.0), a photocatalyst consisting of TiO_2_ doped with metallic waste of a door key, enabled the mineralisation of diclofenac, a medicine also known as Voltaren, in water solely through the use of solar radiation.^117^

#### 3.6.3 Ozonation

Ozone (O_3_) is 13 times more soluble in water than oxygen (O_2_) and can oxidise a wide range of organic and inorganic compounds.^118^ Ozone-based decontamination technologies use ozone in various forms, including gaseous, aqueous, and mist, to disinfect surface, appliances and medical equipment.^118,119^ Under near-neutral or acidic pH, ozone interacts with organic pollutants by targeting carbon (–C=C–) or nitrogen (–N=N–) double bonds. Thus, ozonation is one of the most utilised techniques for disinfection and removal of taste, odours, and colour in drinking water treatment plants. Oxidation by ozone occurs *via* direct reaction with dissolved ozone (O_3_) or indirect oxidation *via* (OH˙) radicals. The extension of both reactions throughout the compound's degradation depends on several factors, such as the contaminant's nature, the ozone dose, or the medium's pH. For instance, direct ozonation reactions are often predominant in acidic mediums (pH < 4), see eqn (11) (ref. 100)

11 3O_3_ + OH^−^ + H^+^ → 2OH + 4O_2_

Indirect ozonation reactions are more likely to prevail at pH > 9, where the ozone decomposition generates hydroxyl radicals that have a greater oxidation power than ozone, resulting in a highly effective treatment process (see eqn (12)–(17)).

12 O_3_ + OH^−^ → O_2_ + HO_2_^−^

13 O_3_ + HO_2_^−^ → HO_2_ + O_3_^−^

14 HO_2_ → H^+^ + O_2_^−^

15 O_2_^−^ + O_3_ → O_2_ + O_3_^−^15

16 O_3_^−^ + H^+^ → HO_3_

17 HO_3_ → OH˙ + O_2_

Therefore, ozonation can be an excellent option for laundry greywater treatment due to the alkaline nature of this type of wastewater. The use of ozone for bleaching and garments decontamination offers additional benefits, including a reduction in detergent usage. This decrease in detergent consumption mitigates the negative environmental impact of the resulting wastewater.^120^ However, a primary challenge in using ozone to reduce organic micropollutants is the creation of potentially harmful by-products. These by-products may exhibit greater toxicity than the original compounds.^121^ Additionally, alkaline conditions can promote fast side reactions that generate hydroperoxyl radicals (˙HO_2_) which in turn limits the oxidation ability, see eqn (18).

18 HO + O_3_ → ˙HO_2_ + O_2_

Various methods can be used to produce ozone from oxygen, such as photochemical (UV), electrical (corona) discharge, chemical, radiochemical, and electrolytic methods. However, ozone rapidly decomposes into oxygen, so it cannot be stored and must be continuously produced by an ozone generator which often consumes large amounts of energy and forms a major obstacle to scaling such processes. Thus, adequate ozonation reactor design can improve the mass transfer of O_3_ from gas phase to the liquid phase to be able to attack the organic molecules chemical bonds and eventually help in enhancing efficiency and reducing energy consumption. Furthermore, the efficiency of ozonation process in wastewater treatment can be improved by adding other oxidation agents such as UV irradiation and hydrogen peroxide.^122,123^

#### 3.6.4 Combination of O_3_/UV/H_2_O_2_

Ultraviolet radiation can degrade pollutants compounds through the photolysis process or by making them more susceptible to hydroxyl radicals attacks. Moreover, combining ozonation with ultraviolet irradiation (O_3_/UV) can help in generating large concentrations of hydroxyl radicals and eventually speed up the decomposition of the organic molecules. Ozone absorbs the UV irradiation and generate hydroxyl radicals in a two-stage process. Firstly, O_3_ goes through photo induced homolysis as shown in eqn (18). Then the oxygen atom O (^1^D) react with water forming hydroxyl radicals, see eqn (19) and (20).

19 O_3_ + UV → O_2_ + O(^1^D)

20 O(^1^D) + H_2_O → 2HO

Nevertheless, the generated hydroxyl radicals can interact with the O_2_ molecule forming hydrogen peroxide, see eqn (21).

21 O_3_ + H_2_O → 2[˙OH] + O_2_ → H_2_O_2_ + O_2_

The hydrogen peroxide in the presence of ozone can go through a series of reactions as follows:

22 H_2_O_2_ → HO_2_^−^ + H^+^

23 HO_2_^−^ + O_3_ → ˙HO_2_ + O_3_^−^

24 ˙HO_2_ → ˙O_2_^−^ + H^+^

25 ˙O_2_^−^ + O_3_ → O_2_ + O_3_^−^

26 ˙O_3_^−^ + H^+^ → HO_3_

27 ˙HO_3_ → ˙OH + O_2_

### 3.7. Nanobubbles

**Fig. 10:**
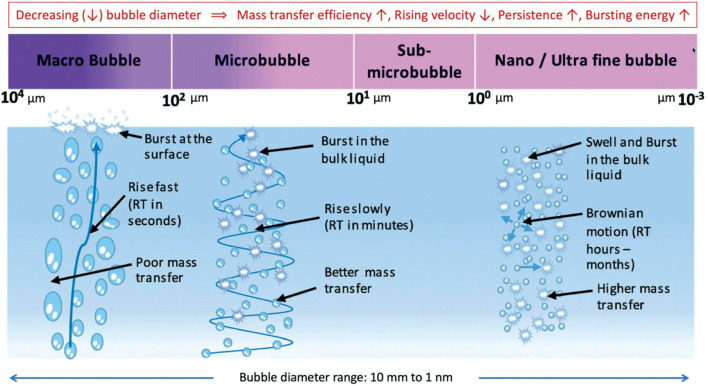
Range of bubbles sizes and corresponding major properties.^125^

Nanobubbles (NBs) are widely used in improving water quality and wastewater treatment due to their unique ability in decomposing and removing pollutants. The term nanobubbles is used to describe microscopic gas bodies “bubble” sized at nanoscale (<1 μm). However, in several studies, NBs are classified on a size scale of less than 200 nm. These bubbles can be produced using different techniques such as the electrical excitation method (electrical cavitation), solvent mixing (exploits gas solubility differences between solvents), shear technique (using rough, porous surface) and nozzle-based nanobubble generators. Each method has its own advantages and disadvantages, for instance, shear methods are highly scalable while other techniques such as nozzle-based techniques can be limited in the amount or the size of the produced bubbles. Furthermore, nanobubbles are neutrally buoyant that can exist in bulk solutions or attached to surfaces.^124^ As shown in Fig. 10, while macro bubbles rise directly and rapidly to the surface of the liquid and burst out, NBs are stable and have no net directional movement but a Brownian motion. NBs can stay for a long time in the bulk liquid which increase their ability to interact with various types of pollutants compounds. NBs have high oxidative ability; when they burst, they generate hydroxyl radicals and cause high mass transfer of gas into the liquid, which can reduce the surface tension and pH of the aqueous solution.^125^

The stability of nanobubbles can be attributed to several acting forces such as the internal gas pressure, bubble surface charge and liquid surface tension. Where the pressure difference produced by surface tension depends also on surface charge density as shown in eqn (28).28
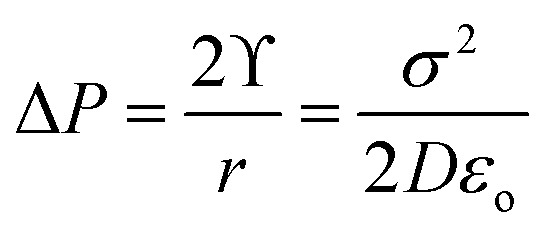
where *ϒ* is the surface tension, *σ* is the charge density, *D* is the dielectric constant, *ε*_o_ is the permittivity of vacuum, and *r* is the bubble radius.^126^

Nanobubbles (NBs) interact with pollutants such as surfactants in two ways: contaminants adhesion and oxidation. In contaminants adhesion, NBs can act as carriers for contaminants where their surface charges attract polarised molecules. Additionally, hydrophobic molecules can be absorbed into the surface of the bubble. Meanwhile, nanobubble oxidation occurs when the NBs collapse and radical oxidative species (ROS) are released by using UV light, sonication, or rapid pressure change.^126^ NBs can act as an anti-biofouling and scale inhibitor where their surface charges attract contaminants such as biofilms or algae and scour them from surfaces. Furthermore, these surface charges can interact with the protein surfaces of microbes, which helps prevent disease-causing bacteria from forming on surfaces.^127^ The high stability of ozone nanobubbles facilitates sustained mass transfer and has the potential to reduce the energy consumption of conventional micro/macro bubble ozonation processes. In traditional methods, a significant portion of ozone is lost in gaseous form due to buoyancy forces and the rapid collapse of larger bubbles.^128^

### 3.8. Hybridised laundry wastewater treatments and recommendations

Dimoglo *et al.*^129^ utilised electrocoagulation (EC) and electro-flotation (EF) techniques to purify laundry wastewater in order to be reused in the washing processes. As a result, 90% of colour, turbidity, and surfactants were removed at an operational current density of 5.26 mA cm^−2^, pH of 5.5, a processing time of 5 minutes, and energy consumption of 1.25 kW h m^−3^. Adding an adsorption process with active charcoal as post-treatment to the EC/EF reactor increased removal efficiency to 97% for turbidity and 95% for colour and synthetic surfactants, reducing energy consumption by 50%.^65^ In another study, EC/EF technique removed 100%, 98%, 94%, and 91% of colour, microplastic, surfactant, and COD from laundry wastewater at an operating cost of 1.32$ per m^3^ when used after filtration as a pre-treatment process utilising sampling net with a mesh density of 26 μm.^130^ Moreover, Nascimento *et al.*^131^ performed laundry wastewater treatment experiments using a hybridised water treatment system, which includes chemical coagulation/flocculation and sedimentation (C/F/S) followed by a microfiltration (MF) process. This experimental system reduced colour by 98.4%, turbidity by 99.1%, surfactants by around 71.7%, COD by 68.6%, total dissolved solids (TDS) by 55.6%, and total organic carbon (TOC) by 56.3%. In a similar study by Huang *et al.*^132^ performed a laundry. wastewater treatment using (C/F/S) techniques, with the addition of activated carbon as an adsorption material before the MF process. The study results showed that adding an adsorption process increased the removal efficiency of turbidity, colour, surfactant, and COD to 99.4%, 99.9%, 92.9%, and 80%, respectively. Based on these studies, it becomes evident that the utilisation of adsorption (AD) technique as a secondary treatment process can enhance the overall effectiveness of physicochemical laundry greywater treatment systems such as coagulation and flocculation, particularly when integrated with additional filtration systems as well as reduce the energy consumption for electrocoagulation (EC) systems. According to Mostafazadeh *et al.*,^63^ electrooxidation (EO) has demonstrated higher efficiency in removing COD and similar effectiveness in reducing turbidity compared to electrocoagulation (EC), where the primary distinction between EC and EO lies in the type of cathode. AOPs can be integrated with membrane filtration techniques to create a photocatalytic membrane reactor (PMR). This reactor concentrates pollutants near the catalyst surface, allowing for their subsequent photocatalytic degradation. The combination of these two technologies can be seamlessly integrated because they share similar operational conditions, simplifying the control of the system.^133^ PMR employs membrane filtration to segregate pollutants in the water, enabling the elimination of rejected pollutants through photocatalysis. This approach offers the additional advantage of mitigating membrane fouling by establishing a self-cleaning membrane. The majority of PMRs utilise a blend of photocatalysis and pressure-driven membrane techniques, including ultrafiltration (UF), nanofiltration (NF), and microfiltration (MF).^134^

## 4. Artificial intelligence (AI) in wastewater treatment

**Fig. 11:**
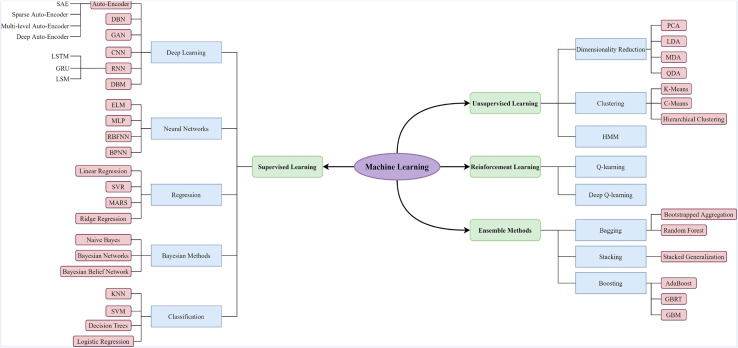
An overview of diverse machine learning techniques.^135^

**Fig. 12:**
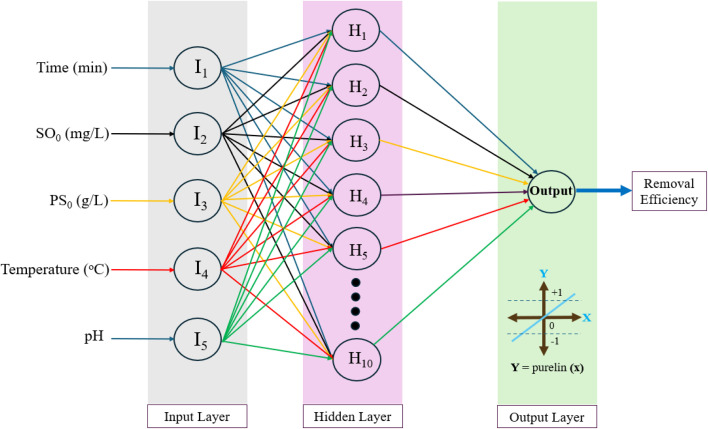
The best artificial neural networks ANN topology used to predict removal efficiency of safranin O (SO) dye and optimise AOPs adapted from Fetimi *et al.*^141^ with permission.

Wastewater treatment is an intricate process, with uncertainties causing variations in effluent quality, costs, and environmental risks. Due to its ability to solve complex nonlinear problems, Artificial intelligence (AI) has evolved into a potent tool in managing and exploring wastewater treatment systems. Machine learning (ML) is a specialised area within the field of AI that focuses on creating and examining statistical algorithms capable of learning from data, generalising to unseen data, and executing tasks without explicit instructions. As shown in Fig. 11, ML is categorised into four main types supervised learning, unsupervised learning, reinforcement learning and ensemble methods In the realm of AI techniques employed in wastewater treatment applications, algorithms predominantly encompass artificial neural networks (ANN), regression algorithms (linear, logistic), support vector machines (SVM), fuzzy logic (FL), genetic programming (GP), random forest (RF), gradient boosting (GB), and search algorithms such as genetic algorithms (GA) and particle swarm optimisation (PSO).^135,136^ However, ANN stands out as the most prevalent and extensively applied model for AI in the domain of wastewater treatment.^137–140^ For instance, Fetimi *et al.*^141^ constructed an ANN model to predict pollutant removal efficiency and optimise an advanced oxidative process (AOP) that involves using heat-activated persulfate to remove the Safranin O (SO) dye. As depicted in Fig. 12, The final ANN topology comprises three layers: a five-node input layer, ten nodes in the hidden layer, and an output layer. This model incorporates five input parameters: process time, initial concentration of SO dye, initial concentration of persulfate (PS), liquid temperature, and initial solution pH. Currently, AI is mainly used to help evaluate the efficiency of pollutant removal, optimise parameters of the treatment systems, and control membrane fouling.^139^

### 4.1. AI in coagulation/electrocoagulation

Coagulation is a fundamental process for contaminant removal in water treatment plants (WTPs). A key challenge in this process is determining the optimal coagulant dosage, which is traditionally assessed using the jar test method. However, this test is time-intensive, expensive, prone to human error, and highly sensitive to fluctuations in raw water quality. AI integration can significantly reduce costs and minimise the time required for experimental jar testing by accurately predicting the optimal coagulant dosage and forecasting water quality under real-world operating conditions.^142,143^ In a study aimed at optimising coagulant dosage in a water treatment plant, J. Kim *et al.*^144^ developed a deep learning model that includes a one-dimensional convolutional neural network (Conv1D) and a gated recurrent unit (GRU) to predict the coagulant dosage and sedimentation basin turbidity. The model utilises eight input parameters, including raw water flow rate, temperature, pH, electrical conductivity, alkalinity, turbidity, total organic carbon (TOC), and pre-chlorination levels, to estimate the required coagulant dosage. Two optimisation approaches were employed: the first involved predicting the coagulant dosage while maintaining sedimentation basin turbidity below 1.0 NTU, whereas the second assessed the effects of systematically reducing the predicted dosage by 5%, 10%, 15%, and 20% on sedimentation basin turbidity. The study's findings highlight the model's effectiveness in optimising coagulant dosage, achieving a significant reduction of approximately 22%.

AI algorithms, including response surface methodology (RSM), artificial neural networks (ANN), and genetic algorithms (GA), are widely utilised to model the electrocoagulation process. These techniques aid in predicting the removal efficiency of pollutants such as turbidity and chemical oxygen demand (COD) while also identifying the optimal operating conditions.^145–147^ Obi *et al.*^148^ modelled and optimised an electrocoagulation (EC) system using Artificial Intelligence (AI) algorithms, including Artificial Neural Networks (ANN), Adaptive Neuro-Fuzzy Inference Systems (ANFIS), Particle Swarm Optimization (PSO), and Genetic Algorithms (GA). Five input variables were considered: pH, current intensity, electrolysis time, settling time, and temperature. Both ANN and ANFIS models showed excellent fit with experimental data (*R*^2^ = 0.9993), with error indices indicating the ANFIS model performed better. Process optimization with GA and PSO predicted a turbidity removal efficiency of 99.39% under optimal conditions (pH 3.1, current intensity 2 A, electrolysis time 20 min, settling time 50 min, temperature 50 °C).

### 4.2. AI in membrane filtration

Membrane fouling stands as a primary impediment in the widespread implementation of membrane filtration technologies. The precise prediction or simulation of membrane fouling behaviours using AI techniques holds paramount importance for comprehending fouling mechanisms and formulating efficient strategies to mitigate fouling.^149^ Park *et al.*^150^ created a deep neural network (DNN) employing *in situ* fouling image data to model the growth of organic fouling and the subsequent flux reduction in both nanofiltration (NF) and reverse osmosis (RO) membrane filtration systems. This DNN model demonstrated high performance, achieving an *R*^2^ value of 0.99 and a root mean square error (RMSE) of 2.82 μm for the simulation of fouling growth. Additionally, it attained an *R*^2^ of 0.99 and an RMSE of 0.30 L m^−2^ h^−1^ for the simulation of flux decline. Jawad *et al.*^151^ performed a study to predict the permeate flux in forward osmosis (FO) using a multi-layered neural network. This study achieved an accuracy of 97.3% utilising a trained ANN encompassing three hidden layers with 25 neurons in the first two layers and 40 neurons in the third layer. Moreover, the study revealed that a higher number of neurons and a reduced number of hidden layers proved advantageous in enhancing the accuracy of ANN. Organic solvent nanofiltration (NF) membranes pose a formidable challenge due to the wide variety of potential solvents and the intricate interplay among the solvent, solute and membrane. Hu *et al.*^152^ utilised ML techniques, including ANN, SVM and RF, to predict the separation performance of an organic solvent nanofiltration process using around 19 input parameters and 38 430 data points. These models exhibited a high prediction accuracy, reaching as high as 98% for permeance and 91% for rejection. The outcomes of this research lay the foundation for standardised data practices, not only for performance prediction but also for enhancing membrane design and development.

### 4.3. AI in the adsorption process

Utilising artificial neural networks enables the mitigation of certain limitations inherent in traditional adsorption models. Particularly in terms of offering enhanced predictive accuracy under varied operating conditions, such as multicomponent adsorption systems, where simultaneous antagonistic, synergistic, and noninteraction adsorption behaviours may manifest.^153,154^ For instance, Tanzifi *et al.*^155^ designed an ANN utilising pH value, initial concentration, adsorption time, and the dosage of polyaniline/SiO_2_ nanocomposite as input parameters. This ANN was specifically developed to simulate the adsorption process of amido black 10B dye and predict the removal efficiency of the dye from aqueous solutions. Remarkably, the 8-neuron configuration exhibited the lowest mean error and the highest coefficient of determination value (*R*^2^) among the tested network architectures. In another study, Afolabi *et al.*^156^ formulated an ANN using MATLAB software to simulate the pseudo-second order Kinetics governing the paracetamol adsorption process using orange peel activated carbon. In this study, the optimum configuration of the ANN includes 18 hidden neurons, hyperbolic tangent sigmoid transfer function (tansig) at inputs layer, linear transfer function (purelin) at the output and Levenberg–Marquardt as the backpropagation algorithm. Moreover, Vakili *et al.*^157^ utilised both response surface methodology (RSM) and artificial neural network (ANN) techniques to optimise the removal effectiveness of four distinct types of organic micropollutants. This adsorption process was carried out using a fixed-bed column packed with cross-linked chitosan/zeolite. Furthermore, Zhu *et al.*^158^ determined that the ML algorithm random forest (RF) emerged as the most effective predictive algorithm when assessing the impact of carbon-based materials on the adsorption of tetracycline and sulfamethoxazole.

### 4.4. AI in biological wastewater treatments

Machine learning (ML) has been employed to discern the essential microorganisms within specific biological units in wastewater treatment. In this context, microbial communities serve as inputs for predicting pollutant removal. For example, Wijaya & Oh^159^ developed distinct ML models using different algorithms such as SVM and RF to predict the operational characteristics of three biological wastewater treatment units, namely a Membrane Bioreactor (MBR), a Sequencing Batch Reactor (SBR), and a conventional activated sludge system. These ML models exhibited an average accuracy exceeding 91.6% and identified *Ferruginibacter* as the keystone microorganism in the MBR system. Moreover, Dutta *et al.*^160^ employed RF algorithms to achieve successful predictions of various phases of sulfidogenesis with an accuracy of 93.17%. Additionally, the RF algorithm demonstrated excellent performance in predicting sessile and effluent microbial communities in a bioreactor, achieving a perfect accuracy of 100%. In a separate study investigating the modelling of water quality in the effluent from an MBR, Zhong *et al.*^161^ used ML algorithms including linear regression (LR), regularised linear regression (RR), kernel ridge regression (KRR), polynomial regression (PR), *k*-nearest neighbour (KNN), support vector machine (SVM), gradient boosting (GB), and random forest (RF). The study focused on predicting concentrations of COD_out_, TN_out_, NH_4_^+^–N_out_, NO_3_^−^–N_out_, and NO_2_^−^–N_out_. The results demonstrated the capability of these algorithms to effectively simulate MBR performance in treating high-salinity wastewater. Integrated learning algorithms, RF and GB exhibited the best fit for effluent quality data.

### 4.5. AI in advanced oxidation processes and hybrid treatments

The primary challenge in advanced oxidation processes (AOPs) and hybrid treatment systems is optimising the operational parameters to enhance pollutant removal efficiency while minimizing energy consumption and operational costs.^100^ Consequently, the majority of AI studies have focused on predicting removal efficiency and identifying the optimal operational conditions. For instance, Picos-Benítez *et al.*^162^ employed an artificial neural network-genetic algorithm (ANN-GA) model to predict the treatment efficiency of wastewater containing bromophenol blue dye through an electro-oxidation (EO) process. The ANN model was trained on data from 51 electrolytic trials, utilising electrolysis time, flow rate, current density, pH, and dye concentration as input variables. The GA determined optimal operating conditions at 10 min electrolysis time, 11.9 l min^−1^ flow rate, 31.25 mA cm^−2^ current density, pH 2.8, and 41.25 mg l^−1^ dye concentration, yielding a discolouration efficiency of 88.8 ± 0.3%. Oviedo *et al.*^163^ performed research to evaluate the photocatalytic activity of nano-zeolite (nANA) in Rhodamine B (RhB) degradation using ML algorithms, including Random Forest (RF), Artificial Neural Network (ANN), and Xtreme Gradient Boosting. The ANN model (3 : 6 : 1 structure) demonstrated the best predictive performance (*R*^2^ = 0.98 for training, 0.9 for testing and RMSE < 5.0), predicting 50.37 ± 1.01% RhB removal at pH 5.7, initial concentration of RhB = 200 mg l^−1^ and ANA = 2.75 g l^−1^ after 180 min under visible light. Yang *et al.*^164^ used multi-output regression random forest (MORF) artificial intelligence (AI) models utilising fluorescence spectra to predict the removal efficiency of trace organic contaminants (TrOCs) during UV/H_2_O_2_ treatment of municipal secondary effluent. The MORF model demonstrated high predictive accuracy (*R*^2^ = 0.83–0.95), enabling its potential application as a rapid-response feedback mechanism for optimising the UV/H_2_O_2_ treatment process. Nghia *et al.*^165^ developed a three-layer artificial neural network (ANN) utilizing logsig–purelin transfer functions to model the removal process of the antibiotic sulfamethoxazole (SMX) from an aqueous solution using an ozone-electrocoagulation hybrid treatment system. The key operational parameters were optimised using response surface methodology (RSM) to maximise the removal efficiency of SMX. The results demonstrated that the optimal conditions are a current density of 33.2 A m^−2^, a reaction time of 37.8 minutes, a pH of 8.4, and an ozone dose of 0.7 g h^−1^, resulting in a removal efficiency of 99.65%. It is worth mentioning that there are no research has been found to date on the implementation of AI in utilizing nanobubbles (NBs) for wastewater treatment.

## 5. Limitations and future trend

### 5.1. AI models limitations and challenges in the real world

AI models can significantly improve the accuracy of wastewater treatment processes by predicting and controlling various parameters. However, it depends on various factors such as the quality of data, the complexity of the treatment process, and how well the AI models are integrated into existing systems.^166,167^ For instance, Xu *et al.*^168^ developed AI models to predict effluent phosphorus levels in a wastewater treatment plant with incomplete influent phosphorus and chemical dosage data. The support vector machine (SVM) model showed moderate accuracy, with an *R*^2^ of 0.637. The long short-term memory (LSTM) model, which predicted phosphorus load one day ahead, had a lower *R*^2^ value of 0.496, indicating limited predictive performance due to the lack of complete data. In contrast, AI models have been shown to achieve up to 100% accuracy in predicting sessile and effluent microbial communities in a bioreactor when trained on high-quality data.^160^ There are several limitations and challenges in deploying these AI models in real-world wastewater treatment facilities. The first challenge is the availability of high-quality data. Wastewater treatment data exhibits significant variability influenced by factors such as influent composition, weather, operational changes, and poor data collection systems. AI models, typically trained on more stable datasets, struggle to account for this uncertainty and fluctuation. Moreover, AI models often rely on large datasets, some of which may contain sensitive information. Ensuring data security and privacy, particularly when it pertains to specific geographic locations or proprietary treatment processes, presents a significant challenge and requires robust protection measures.^167^ The scalability of AI solutions can be limited since an AI model trained on historical data from one treatment plant might not generalize well to another facility with different operational parameters or environmental conditions. Additionally, Wastewater treatment processes involve numerous physical, chemical, and biological interactions. Modelling these complex interactions accurately is challenging, as AI models may struggle to account for all the variables that influence treatment outcomes. The integration of AI models with legacy systems in wastewater treatment plants presents technical and financial challenges, often necessitating substantial system upgrades or replacements. These difficulties are particularly pronounced when deploying AI models in real-time operations, as wastewater treatment processes need to be responsive to changes in water quality. This can involve significant upfront costs, including the installation of sensors and data infrastructure which can be challenging for smaller treatment plants. Furthermore, wastewater treatment facilities must meet stringent regulatory standards for discharge quality. Automated AI models must be able to comply with these regulations, which often require strict monitoring and reporting. However, maintaining and updating implemented AI solutions can be a resource-intensive task, as AI models require continuous monitoring to ensure optimal performance. And require personnel with specialized knowledge in both wastewater treatment processes and machine learning to evaluate model performance against real-world data, identifying deviations, and making necessary adjustments to sustain accuracy and reliability.^169^

### 5.2. AI in laundry greywater and future trend

Future research is expected to continue to focus on advances in detecting and identifying emerging contaminants. For instance, Zhu *et al.*^170^ devised a deep learning convolutional neural network architecture known as PlasticNet to enhance the recognition of microplastic (MP) types. This approach addresses challenges related to the presence of additives, adsorbed contaminants, changes in thickness, and surface modification when analysing MP samples using FTIR spectra. Therefore, AI can be used to predict changes in microplastic pollution levels in response to variations in laundry operating conditions. Factors such as different concentrations of detergents and bleaches, water temperature, and the number of washing and drying cycles can be assessed for their impact on the release of microfibers from garments, including materials like polyester, nylon, polyamide, and acrylic. Additionally, AI can be used to assess the quality of the treated laundry wastewater. For example, Abuzir & Abuzir^171^ formulated three machine learning models designed for water quality classification, explicitly distinguishing between potable and non-potable water.

Laundry greywater exhibits variations in pollutant compositions and concentrations, contingent upon the specific sources generating the wastewater. Consequently, AI can encounter challenges, including the scarcity of available data and the identification of key parameters and indicators essential for the development of accurate AI models in this context. Using IT technologies such as cloud computing and the Internet of Things (IoT), in combination with AI, can play a crucial role in collecting laundry greywater data, remotely controlling greywater treatment systems, and performing various other functions to streamline and optimise the wastewater treatment processes. For instance, Kakkar *et al.*^172^ developed a water quality monitoring system for the residential water tank. This system integrates IoT and AI, incorporating physical and chemical sensors to detect parameters such as pH, turbidity, colour, dissolved oxygen, and conductivity. In case any of the parameters fall below predefined standard values, the system triggers an alarm notification to alert the user. This proactive approach enables users to anticipate water pollution in their home tanks.

Future research is anticipated to employ AI algorithms for the development of novel photocatalysts aimed at improving greywater treatment, where a mechanistic understanding of catalytic organic reactions is essential for designing advanced catalysts, exploring reactivity, and promoting sustainable chemical processes. As an example, Burés and Larrosa^173^ developed a novel artificial intelligence (AI) tool capable of classifying chemical reaction mechanisms using concentration data. This tool can make predictions with an accuracy of 99.6%, even when dealing with realistically noisy data. Morover, Burger *et al.*^174^ employed a mobile robot, guided by a batched Bayesian search AI algorithm, to autonomously explore improved photocatalysts for hydrogen production from water. This autonomous optimisation process identified photocatalyst mixtures with sixfold higher activity compared to initial formulations by selectively incorporating beneficial components while eliminating detrimental ones.

Furthermore, efforts will continue to advance environmentally sustainable, renewable, and cost-efficient separation media and innovative treatment systems specifically designed to target specific micropollutants. These endeavours may include scaling up nanotechnology applications, such as employing metal oxide nanoparticles as adsorbents, integrating nanobubbles (NBs) into membrane filtration processes to mitigate fouling, and optimising ozonation processes to enhance overall efficiency.

## 6. Conclusions

This review provides a comprehensive analysis of laundry greywater treatment processes to mitigate the escalating risks associated with the variation in quality levels of laundry wastewater discharged into the environment by exploring the opportunities of using AI techniques in this context. Due to its low concentration of pollutants, laundry greywater exhibits significant potential for recycling and can be repurposed as service water for applications such as irrigation, toilet flushing, and additional washing activities. The choice of technology or method for recycling laundry wastewater is contingent upon the characteristics and types of the associated pollutants. Electrocoagulation has shown improved effectiveness in treating synthetic dyes and organic compounds. When combined with other oxidation treatments like ozonation (O_3_) and hydrogen peroxide (H_2_O_2_), UV irradiation can enhance the removal efficacy of pharmaceutical antibiotics from laundry wastewater. Using a membrane bioreactor (MBR) can be effective in removing microplastics (MP). However, the biodegradability of microplastics is influenced by several factors, including particle morphology and the chemical composition of microplastics. Employing efficient pretreatment with a focus on developing green, renewable, cost-efficient separation media such as superhydrophobic geopolymer foams and nanocellulose hydrogel films is essential to enhance microplastic removal efficiency. Adsorption (AD) as a secondary treatment process can significantly improve the overall effectiveness of physicochemical treatment systems for laundry greywater. This enhancement becomes particularly evident when integrated with additional filtration systems and helps reduce the energy consumption associated with electrocoagulation (EC) systems. Iron oxide nanoparticles (IONPs) have gained popularity as adsorbents due to their large surface area, ability to modify their surface properties to target specific pollutants, and ease of retrieval using magnetic-based separation techniques. Advanced oxidative processes (AOPs) demonstrate considerable effectiveness in eliminating non-biodegradable contaminants. However, challenges such as high energy consumption, chemical inputs, catalyst support corrosion, suboptimal light utilisation, limited ozone mass transfer, and operational costs often restrict their widespread adoption in wastewater treatment. AOPs can be combined with membrane filtration techniques to form a photocatalytic membrane reactor (PMR). PMR utilises membrane filtration to separate contaminants in water, facilitating the degradation of rejected pollutants through photocatalysis. This integration provides the added benefit of reducing membrane fouling by establishing a self-cleaning membrane. Nanobubbles (NBs) can function as anti-biofouling and scale inhibitors by utilising their surface charges to attract contaminants such as biofilms or algae, effectively removing them.

AI is primarily employed to evaluate the efficacy of pollutant removal, optimise treatment system parameters, and control membrane fouling. Artificial Neural Network (ANN) models have been explicitly employed to predict the removal efficiency of Advanced Oxidation Processes (AOPs) targeting specific pollutants like synthetic dyes, thereby optimising the overall process. Similarly, ANNs mitigate certain limitations inherent in traditional adsorption models. These ANN models excel at capturing nonlinear relationships, which are prevalent in multicomponent adsorption scenarios where interactions can be antagonistic, synergistic, or non-interactive. Machine Learning (ML) models are widely applied to identify primary microorganisms within biological treatment units. These microbial communities are then leveraged as inputs to forecast the efficacy of pollutant removal. Moreover, crucial ML algorithms like GB, RF, and DNN have been successfully applied to simulate MBR performance and model/predict membrane fouling behaviour under different conditions. Future AI research is anticipated to prioritise advancements in detecting and identifying emerging contaminants like microplastics (MPs), evaluating the treated laundry greywater quality for reuse purposes, and utilising IT technologies such as cloud computing and the Internet of Things (IoT) for data collection to train AI models.

## Abbreviations

AC: Activated carbon
AI: Artificial intelligence
AOPs: Advanced oxidation processes
ANN: Artificial neural networks
BOD: Biochemical oxygen demand
BiAS: Bismuth active substance
CMC: Critical micelle concentration
COD: Chemical oxygen demand
CTAB: Cetyltrimethylammonium bromide
CSTR: Continuously stirred tank
DHS: Downflow hanging sponge
EC: Electrocoagulation
EO: Electrooxidation
FL: Fuzzy logic
GA: Genetic algorithms
GAC: Granular activated carbon
GB: Gradient boosting
GO: Graphene oxide
GP: Genetic programming
HRT: Hydraulic retention time
IONPs: Iron oxide nanoparticles
IoT: Internet of things
KNN: *K*-nearest neighbour
KRR: Kernel ridge regression
LAS: Linear alkylbenzene sulfonate
LR: Linear regression
MBAS: Methylene blue active substances
MBBR: Aerobic moving bed bioreactor
MBR: Membrane bioreactor
MF: Microfiltration
ML: Machine learning
MORF: Multi-target regression random forest
NBs: Nanobubbles
NF: Nanofiltration
NPEOs: Nonylphenol ethoxylates
N–N: Nitrite/Nitrate
O&G: Oil and grease
PCP: Personal care products
PET: Polyethylene terephthalate
PMR: Photocatalytic membrane reactor
PR: Polynomial regression
PSO: Particle swarm optimisation
purelin: linear transfer function
RF: Random forest
RMSE: Root mean square error
RO: Reverse osmosis
ROS: Reactive radical oxidative species
RR: Regularised linear regression
RSM: Response surface methodology
SBR: Sequencing batch reactor
SBBR: Sequencing batch biofilm reactor
SDS: Sodium dodecyl sulphate
SMBR: Submerged membrane bioreactor
SRT: Sludge retention time
SVM: Support vector machine
TA: Alkalinity rate
TAMR: Thermophilic aerobic membrane reactor
tansig: Hyperbolic tangent sigmoid transfer function
TDS: Total dissolved solids
TFS: Total fixed solids
TEAQ: Dihydrogenated tallowethyl hydroxyethylmonium methosulfate & ditallowethyl hydroxyethylmonium methosulfate
TH: hydrotimetric rate
TKN: Total Kjeldahl nitrogen
TN: Total nitrogen
TOC: Total organic carbon
TP: Total phosphorus
TrOC: Trace organic contaminant
TS: Total solids
TSS: Total suspended solids
TVS: Total volatile solids
TX-100: Octylphenolethoxylate
UASB: Up-flow anaerobic sludge blanket
UF: Ultrafiltration
UN: United nations
UV: Ultraviolet
WHO: World health organisation

## Data availability

No primary research results, software or code have been included and no new data were generated or analysed as part of this review.

## Conflicts of interest

There are no conflicts to declare.
